# Extracts

**Published:** 1882-04

**Authors:** 


### Extracts.

---

Quackery in the Profession.—The profession is probably unaware of the progress steadily made by medical quackery in its diverse forms and disguises. Quackery which is not medical—in the sense of being practiced by duly qualified men—is undoubtedly an evil, but its consequences are not comparable with the effects of such quackery as is growing apace within our own ranks, and slowly it may be, but surely, undermining the respect and confidence which the profession has hitherto deserved and received from the public. We sometimes wonder that our calling does not command the warm recognition in certain quarters to which it seems entitled. For a sufficient explanation of this default in the estimation of society, let us look to the prevailing and almost daily increasing popularity of "systems" and "cures" tacitly, if not avowedly, supported or countenanced by the profession. There is a sentimental and mock-heroic spirit abroad which burlesques the candor of "truth-seeking," and even mimics the impulses of chivalry. We hesitate to condemn any system, "lest there should be some good in it," and we are too tender-hearted and polite to deal honestly by its promoters, even though we recognize the fallacy of their pretensions, and more than suspect their motives. This is not a faithful line of conduct in reference to our profession, nor is it loyal to science, which is one of the many constitutent parts and aspects of truth. We know, or ought to know, that a perfectly just and truthful conception of the science of medicine must bar the recognition of *systems* and *cures* of any class or description. The art of healing is not a system, and can never be made one. It is simply an intelligent application of the laws of health in the remedy of disease. We study the "symptoms" of a malady with a view to the acquisition of precise knowledge as to its nature, course, and rational treatment. We pursue the investigation of disease over the boundary-line of death, and explore the cadaver with a view to ascertain the effect of the morbid state on the organism and to elicit its organic causes, albeit we too commonly confound effects with causes. We test the powers and analyze the constitution of drugs, and we scrutinize and make careful trial of methods of treatment, to obtain a reasonable acquaintance with their natures and actions. In brief, we take any amount of trouble and resort to every means at our disposal to render the principles and practice of our art *rational.* This is our duty, and it is the only method consistent with self-respect and professional integrity; but, if side by side with this policy, we cherish a spirit of credulity which renders us ever ready to countenance systems of which we can know nothing—because there is nothing to know—and take a false pride in showing friendliness to quacks and charlatans, the good work we ourselves may do is changed to evil by reason of the actual or implied sanction we give to the bad work done by others. Nothing is so much needed just now as the rise in our midst of a stern and uncompromising apostle of sincerity in science—a man of unpitying animosity to humbug in all its forms, who will not hesitate, at any bidding, to denounce wrong-doing and untruthfulness, let who may be the offender. It is time that a spirit of manliness went out in our midst to chase away the lying spirit of mock courtesy—the faint-hearted and time serving sentimentality—which makes us so ready to look kindly on any pretender, and so reluctant to expose any pretense.

There cannot possibly be a "system" or "cure" in medicine. There are no rule-of-thumb methods and no *mysteries* in true science. If we do not know what a remedy is, and how it acts, we have no right, as honest men, to employ it. The time has passed for the working of cures by charms and the recourse to nostrums. We pander to the credulity of the unskilled community when we show ourselves credulous. We patronize and encourage quackery when we extend professional recognition to a quack. Every man is a quack—whether qualified or unqualified—who employs a remedy without knowing why, or who adopts a "system" in medicine. The profession must speak out clearly and strongly on this point, and without delay. From the highest places in the society to the lowest ranks of the people, there is just now a grievous readiness to "believe in" quacks and quackery. We have ourselves to thank for this most adverse "feeling" and "influence." It is the stirring of the viper we have brought in from the cold, where physicians and surgeons of more robust intelligence than those of to day left it—the viper we have warmed and fed and brought back to life; and now it is preparing to rise and sting the hand that caressed it. The way to encounter the charlatanry which is making head against science is to be at once more candid and more conspicuously *honest* in our dealings with the public. We must lay aside the last vestige of the robe of mystery, and show by our words and works, our conduct and policy, that medicine is not a science that admits of inspiration, and that the practice of healing is not an art which can be acquired by the unlearned. There is no system or cure, or charm or nostrum, known to the profession; our calling consists solely in the rational study and treatment of disease on common-sense principles. For those who pretend to a sort of inspiration we have no professional friendship; and towards the promoters of systems and 'pathies we can have no leaning, or any feeling other than that of suspicion, if not pity and contempt. They can have no place in our professional intercourse, and we can have nothing to say to them or their work. This is the only sentiment worthy of the medical profession in its dealings with medical quacks, and the time has come when the revival of its old spirit is most earnestly to be desired.—*Lancet.—Medical News.*

---

Spinal Curvature.—Our next case is this little fellow, aged 5, whose name is Thomas Dillon. The parents are both healthy, and the child was strong until the last two years, when he has had intermittent fever. The first symptoms of pain in the back and difficulty in stooping were noticed eight months ago. He was brought to me yesterday by Dr. Joyce, of this city. I now throw my key on the floor, and observe how he picks it up. He does not stoop as children ordinarily do, but keeps his trunk erect and places his hands on his knees. I place him on a stool and ask him to jump to the floor, and you observe that he does so with his hips, knees and ankles all bent, which would indicate trouble of the spine and lead us to a further examination. Stripping him naked, I now place him on my lap, with his face downward, legs hanging over one thigh and arms over the other, and gradually open my legs. You see I do not give him pain, even as I press together his head and buttocks. As I make pressure of the eleventh and twelfth ribs of the left side against the vertebræ, I cause pain, and he has reflex muscular spasm as evinced by his legs. As I make pressure on the point of deformity over the spinous processes, he experiences no pain. This is a point of great importance for you to know. The books say there will be pain on pressure over the spinous processes of the diseased vertebræ, but you see he feels no pain on pressure at that point, and only on pressure from the side. We now apply a very closely-fitting woven shirt and place the "dinner pad" under the shirt. It is important that the patient should previously have had a good dinner. The child is now placed in the suspending apparatus, and gentle traction is made to the point of comfort. Now, does this make you feel any better? Tell me, my boy, when you are *comfortable.*

Gentlemen, you now see the danger of placing patients under chloroform during suspension, as advised by Langenbeck. How would I know when to stop if they were anæsthetized? Under no circumstances, gentlemen, will you call this Sayre's method, for I do not wish to be responsible for the murders that may be committed by it.

You now observe that this amount of suspension gives pain, and this does not. You see the least difference makes pleasure or pain. [The doctor then varied the extension to suit the comfort of the child.]

In placing your bandages in the vessel of water always put them on end and not on their sides. As long as the gas escapes it shows that all the plaster is not moistened, but as soon as it ceases, there is evidence that all the plaster is saturated. If placed on their sides, the gas cannot escape, and, consequently, the plaster will not be wetted through the bandages.

As the bandages are now applied you see they must not be drawn too tight. It is important, also, that the patient should *remain* quietly suspended at the exact point of comfort during the whole application. "Now, how long can you remain so?" "Oh, a good while," the patient answers. You observe I press the layers of the bandages well together and stroke them with my hands so as to prevent any folds or pleats, as we wish to have the surface perfectly smooth. The plaster above the crests of the ilea is well rubbed in to obtain a firm base of support, and pressure is thus made at the sides to relieve the weight from the bony projections. The patient is now laid upon the bed so the plaster may dry. We afterwards remove the "dinner-pad" and cut the jacket down under the arms, and also at the lower margin in front, that he may flex his thighs to sit down, and we thus make it perfectly comfortable.

It is important for you to get crinoline, or cross-barred muslin, as the best material for your bandages. With other cloth you will make failures. Cheese cloth, for instance, is too loose. You should procure crinoline that has no sizing, or glue, in it, as this is a frequent cause of the jackets "breaking down," or becoming soft and yielding like India rubber. The crinoline should be torn into strips three yards long by two and a half or three inches wide, according to the size of the patient upon whom it is to be used. Its meshes may be completely filled by drawing the bandage through very fine and freshly ground plaster of Paris that has not long been exposed to air—the plaster at the same time being rubbed into the material.

"The seamless skin-fitting shirts," manufactured by the Bickford Knitting Company, of No. 689 Broadway, are the shirts used by me. They, however, are not essential, as anything that will answer the purpose may serve, as I once used stockings that I had divided and sewed into a shirt. For instance, many think the tripod absolutely necessary in the treatment. This is not the case by any means, as a pulley secured in a wall or beam may be of equal service. Children are often brought to me with their stomachs so distended with gas that I have to cut out a piece of the jacket after the flatulent condition is relieved. If in this condition, it is generally better to place them in the horizontal position for a few days till digestion is improved.—*Clin. Lecture by Lewis A. Sayre, M.D., in New England Med. Monthly.*

---

ABDOMINAL SECTION FOR INTESTINAL OBSTRUCTION.—Perhaps the greatest development in abdominal surgery, in the near future, will be in the direction of the treatment of intestinal obstruction. Very many of these cases are at present allowed to die without any attempt being made to ascertain or to remove the cause. And, it happens that up to now, little has been done or written upon the subject of opening the abdomen for obstruction, so that when it has fallen to the lot of a few operators to have had a large number of cases for observation and treatment, there will rise up an entirely new experience, and a literature of its own, for our future guidance. I look upon it that as much, if not more, has to be learnt in this department of abdominal surgery than in any other, and that a field is open, which will yield a most fruitful harvest of successful results to those who will be fortunate enough to have a sufficient number of instances to observe from.

Intestinal obstruction is often divided into two classes, according as the large or small intestine is the seat of the obstruction; and this is quite a natural division of the subject, inasmuch as the symptoms, the subjects, the nature of the obstruction and the treatment, will each speaking generally, be found to differ in the two classes It is when the seat of the obstruction is in the small intestine that we may look for the greatest aid in exploring the abdominal cavity, since it has been found that the part most often strangulated, either by loops, bands or adhesions, is the lower portion of the ileum, although the colon is sometimes constricted by old inflammatory bands; and the same applies to twists, which are more frequently found in the small intestines. Dr. Habershon says that the sigmoid flexure, especially in old persons, where there has been previous constipation, will bend upon itself, and fall over into the pelvis. Again, cases of intussusception, involving prolapsus of the ileum into the cœcum, and obstruction due to the character of the contents of the bowel, will be more frequently found in the small intestines. Fæcal accumulation of itself rarely, if ever, causes fatal obstruction, though, as Dr. Habershon, remarks, death may arise from the violent remedies employed, as from strong purgatives, or as when the injection of very large quantities of fluid have been followed by a fatal collapse. A gall stone may be the cause of the obstruction, and here is a field open for successful surgical aid. I have myself met with one case where, *post mortem*, a large gall stone was found to be the cause of the fatal illness, being impacted in the jejunum; and Mr. H. R. Ker has recently, before the Midland Medical Society, read the interesting details of a case where he opened the abdomen, searched for and found the stone, opened the intestine, and extracted it. This case, unfortunately, was not a successful one, so far as the patient's life was concerned, but it will tend to show what may be expected to be gained in this direction when the operation is undertaken earlier, and when more perfect means of uniting the severed edges of a wound in the intestine are attained. There is nó reason to suppose but that many similar cases to that one under Dr. Ker's care, will, ere long, be successfully treated.

The symptoms, therefore, from which we should predicate that most benefit would accrue from an abdominal section would be early vomiting, moderate and central distension of the abdomen, absence of urination, and the character of the vomited matters being rather bilious than fæcal. In the operation, it may be said to be best to try first to find the seat of obstruction on the right side of the abdomen, because the ileum is the part of the intestine most frequently involved, and a gall stone would generally be found high up in the duodenum or the upper portion of the jejunum; also, because an intussusception would occupy this side.

In the case of there being found more than one constriction in the calibre of the intestine, the propriety of excising a portion, more or less long, of the intestine in its entirety, as recommended by Kœberle, is worthy of consideration.

It may be found, after making an abdominal section, that the seat of disease is in the large intestine, *e. g.*, cancerous disease of the sigmoid flexure, and that a colotomy would have been the better proceeding. Under such circumstances the opening in the left loin may be at once proceeded with, and it may be regarded that the abdominal section will be found not only to have done no harm, but that it is a preliminary which has led to a more accurate diagnosis, and has also been an aid for the safer and easier performance of the colotomy.—*Thomas Savage, M.D., in Birmingham Med. Review.*

---

CAN A MAN HAVE SYPHILIS TWICE ?—The *British Medical Journal* reports the following valuable remarks by Mr. Jonathan Hutchinson, on this interesting topic:

The man whom we have just seen offers a remarkable example of the occurrence of a second chancre soon after the first. His second sore has been, as I have repeatedly demonstrated, characteristically indurated. He is quite candid, and makes no doubt that his sore was the result of contagion. Yet it is barely a year since he had his first chancre, and this was followed by an eruption, of which he had scarcely got clear when this second sore occurred. The case is proof that a man may have an indurated sore on the penis within a year of a former one, but it is not proof that he may have syphilis twice, for this patient has not as yet had any constitutional symptoms as the result of the last chancre. If, however, you ask me for an answer to the general question, Can a man have true, complete syphilis twice? then I must reply clearly that he can. Such cases are rare—as rare, perhaps, as examples of second attacks of small-pox—but they do occur. I am at present attending a gentleman who has a terrible phagedenic chancre and rupial eruption, and who unquestionably had complete syphilis, chancre, sore-throat, and rash, seven years ago. I have also a second case under care, very much milder, but illustrating exactly the same fact, with almost precisely similar dates. Second chancres are, however, far more common than second attacks of constitutional syphilis. Many of them are the result of fresh contagion, but seem to have no power to produce constitutional symptoms; but others are not from contagion at all, but form in connection with a taint still remaining from the first attack. It is a most important fact that indurations may form in the penis, in every respect exactly like Hunterian chancres, not distinguishable in any way, and yet they may be merely recurred sores, and the products of constitutional taint. I have seen this over and over again; and M. Alfred Fournier, of the St Louis Hospital, has written a very instructive paper on this form of sore. In the case of our patient, it is obviously impossible to say, after the statement which I have just made, whether or not his present sore is the result of fresh contagion. It may simply be a relapse, or it may be a gumma. He, however, confesses to exposure; and, as the sore followed in due course, it is probably true that he was afresh inoculated. Second attacks of syphilis are sometimes, as in the case just mentioned, very severe. The same has, I believe, been occasionally noted in recurred attacks of variola. As a rule, however, they are mild, or even abortive. Third attacks may even occur; and so may, as we are told, third attacks of small-pox. We must explain such facts, I expect, by reference to individual peculiarity and idiosyncracy, but it is important that they should be known. The belief that syphilis can occur but once in a lifetime is very widely spread among a certain class of the public. I have watched with amusement the change in expression in many a young gentleman's face when he got my reply to his smiling suggestion —"A man cannot, I suppose, have the disease a second time?"—*Med. and Surg. Reporter.*

---

Inoculation for Pannus.—The word pannus means a red cloth. In the more severe forms, such as pannus crassus, the resemblance may be easily traced. Pannus in ophthalmology means a vascular keratitis or vascular inflammation of the cornea. We have the pannus crassus, pannus tenuis, traumatic pannus, etc., these divisions depending upon the extent of the inflammation and its causes. Where the vascularity is very slight, or involving only a small portion of the cornea, it may be called pannus tenuis; when the vascularity extends into the layers of the cornea, and the vessels are very numerous, we call it pannus crassus. If the pannus has for its cause inverted cilia, it is termed traumatic pannus.

The most common cause of pannus is granular ophthalmia, where the rough granulations by their continued rasping of the cornea produce the vascular keratitis. It very often occurs in relapsing phlyctenular keratitis, especially when the phlyctenulæ are near the center of the cornea. In entropion, where the lashes brush against the cornea, pannus is often the result. Whatever the cause be, the first indication is to remove it. In many cases after this is done the pannus disappears but again in many it persists. In these cases many remedies have been advised. Argent. nit., cupri sulph., acid tannic, and poultices, besides operative procedures, are a few only of the correctives recommended. These may give some relief in the milder cases, but where you have a true pannus crassus to deal with I do not believe any thing will yield the result that inoculation with blennorrheal matter will.

This mode of treating pannus is given in works on ophthalmology as the *dernier ressort*, and is spoken of as very bold. I shall myself in future in such cases, when both eyes are involved, make it my primary resort. I believe in such cases, where the cornea is highly vascular, that such a procedure—under proper care—is entirely admissible and proper. I do not think it bold. With the cornea in such a condition it is not at all likely to ulcerate and break down. The vascularity is such a safeguard that a bad result is nearly impossible.

Of course when but one eye is involved much caution will have to be exercised. It will be better to bandage the opposite eye, especially during sleep. Should the matter get in the good eye, the disease can be easily aborted by dropping into it a solution of nitrate of silver grs. xx or grs. xl to ℥ j of aqua dest., and the use of iced cloths.

In conclusion, then, I believe in pannus crassus there is but one remedy, and that is inoculation by blennorrheal matter; that the treatment is almost harmless; and that there is no estimate of the good resulting.

Gonorrheal matter, or the discharge from a case of purulent ophthalmia, or that from a case of ophthalmia neonatorum, can be used. Whatever the source, be sure it is pure. Exercise the proper caution, and I am sure you will not be disappointed.—*W. Cheatham, M.D., in American Practitoner.*

---

SHALL THE POWER TO LICENSE PHYSICIANS AND SURGEONS BE VESTED IN A STATE BOARD OF EXAMINERS?—Dr. Jacobi, in his closing address to the State Medical Society, has again called attention, among other things, to the subject of Medical Education and licensing to practice. This has been the favorite theme of discussion in medical associations for the last forty years or more, and especially from the time of the organization of the American Medical Association in 1848.

The existence of a great and growing evil is universally recognized and deplored, and many remedies have been suggested. The colleges have it in their power to remedy the evil at once and completely; but appeals to them have been hitherto almost wholly without result. They have, it is true, in many instances, greatly improved their means of instruction, but they have not altered one whit their standard of graduation or qualification to entitle the candidate to a license to practice. It is as easy for a candidate to obtain a license whose knowledge of medicine and surgery is little more than that of educated nurses as for the most accomplished scholars. The same college that sends out our thoroughly educated doctor, whose education has cost him much time and money and hard work, sends out on the same day and with the same honors fifty unqualified men to enter into competition with him. Harvard is the only college that has made a serious attempt to do its full duty in this matter. Situated in the heart of the most highly educated portion of the United States, with 50 per cent of its candidates holding the degrees of A. B. or A. M., it still rejects a larger proportion at its final examination than any other college in the United States. No doubt she is trying honestly to do her duty; but so far she stands alone, and will for a long time to come.

The American Academy of Medicine, organized solely for the purpose of remedying this evil, has thus far inclined to *permanent college endowments* as the most likely to accomplish the end in view.

Latterly some gentlemen, including Dr. Jacobi, have believed that all might be accomplished which is desired by separating the power to grant licenses from the business of teaching, that is, by the establishment of a State Board of examiners, upon whom alone the power of conferring license shall rest. This is the European plan, and this is not the first time it has been suggested by American writers, as the only alternative remaining to us.

In 1878, Dr. B. L. Hovey, of Rochester, in an address delivered before the Alumni Association of the Medical Department of the University of Syracuse, recommended that a central or State Board, shall alone exercise the privilege of examining and licensing for practice in this State.

The difficulties are: Will the colleges relinquish their chartive rights in this respect? Can they be compelled to? If such a board is organized, who shall appoint? If the Government, why will it not become a political appointment? What guarantee that proper men will be appointed?

In our highly democratic government, it is likely to prove a failure, and we shall have to go back to the idea of the American Academy of Medicine: *Endowed* Colleges, or trust alone, as heretofore, to the consciences of those who manage our colleges.

A state board of examination will have to admit representatives from homœopaths and eclectics, both of whom have medical colleges, and under the late laws, are "regular" physicians. Much better have it as it is.—*Editorial in Medical Gazette*

---

Acne Vulgaris (*pustulosa.*)—Our next case is that of a girl, seventeen years of age, in manifestly low condition of health, who exhibits an unusually marked case of acne of the face, back and chest. She has had the disease eight years; its first appearance being slight, she says, but being as bad as it is now for four or five years. It is characterized by large papules and pustules, covering nearly every portion of the face. You can hardly discover a single healthy square line on its surface. The pustules are suppurating freely, so that the face is dotted over with yellowish points. On the back and shoulder-blades the eruption is scarcely less severe. Here you find papules in every stage of development; small and reddish in their commencement, increasing in size as they become older, some of them finally tubercular or suppurating; and scattered about among them are violaceous and muddy-colored spots showing where former papules have existed. The front of the chest over the sternum and upper ribs is quite thickly studded with similar lesions.

Inquiring into her history we learn that the girl has suffered from epilepsy in a mild form since she was three years of age. She has been under treatment for this latter disease many years, taking bromide of potassium most of the time. Now, as the continued administration of this drug often provokes an eruption of acute acne, we are led to suspect this to be the exciting cause in the present case. On further inquiry, however, we learn that the acne had been established before she began to take the bromide. Moreover, judging from the prescription she shows us, the amount she has been in the habit of taking is not sufficient to have produced the acne. It is, then, an idiopathic case of acne vulgaris, probably aggravated by the use of the bromide of potassium.

It will require six months, or longer, to effect a cure. Her low, ill-nourished condition indicates a constitutional treatment. We will prescribe a preparation of iron and quinia, to be taken three times a day. We will also give her, if she continues to take the bromide of potassium, two or three minims of Fowler's solution of arsenic, daily. This will be to correct the influence of the bromide on the acne. If she does not take the bromide, the arsenic will not be given. Locally, the diseased parts must be bathed in hot water, by means of a sponge, fifteen minutes every night. The pustules must be opened, both before and after bathing, either with a needle or sharp pointed knife. After the bath an ointment of precipitated sulphur, a drachm to the ounce of cosmoline, must be thoroughly applied. This treatment will be continued two weeks.

At the end of that time the condition will be very much improved. The strength of the ointment will then be increased to two drachms of the sulphur to the ounce. After continuing with this three or four weeks longer, the ointment will be suspended, and, perhaps, the hot bath also. We will then, possibly, use the following lotion:

℞.—Zinci sulphatis,

Potassii sulphureti, - aa. ℨj

Alcoholis, - - - - f℥ss

Aquæ, - - - - f℥vss. M.

Sig.—To be dabbed on every night and allowed to dry. Shake before using.

Her bowels must be kept open, if necessary, by a saline laxative, taken before breakfast. The tonics will be employed for some time, and, perhaps, later in the treatment-arsenic internally.—*Clinic of Louis A. Duhring, M.D., from Med. and Surg. Reporter.*

---

EXPLOSIVE MIXTURES.—Medical men but rarely pretend to be good chemists, for it would require longer devotion to chemistry than the average medical student can afford; thus can it be marveled at when we see formulæ and prescriptions that, if dispensed according to the wishes of the prescriber, would result in an incompatible combination and often explosive compounds? Thinking it not ill-placed to, perhaps, refresh the memory of the profession regarding such mixtures, especially explosive mixtures, we have selected some examples and formulæ that when combined in certain proportions become dangerous, and in many instances have ended seriously; they are examples that have been experimented with, some intentionally, while others were prescribed by a badly informed physician and dispensed by a very incompetent druggist:

1. Chlorate of potash, powdered galls, tannic acid. M. Ft. Pulvis.—To be used as a gargle. The powders should be mixed separately, with water, and not rubbed together.

2. Chlorate of potash and pulv. catechu.—This combination is intended as a dentifrice. It however should not be dispensed alone. If other combinations are made the danger is averted.

3. Chlorate of potash, hypophosphite of soda and water.—If the salts are rubbed together they will explode, but if dissolved separately in water, and mixed, no harm results.

4. Chlorate of potash, tannic acid, glycerine and water. If the tannin, chlorate of potash and glycerine are rubbed together an explosion ensues, but if the acid is first dissolved in the glycerin and the chlorate of potash in the water and mixed, no harm follows.

5. Chlorate of potash, tr. ferri chlor. and glycerine, half an ounce of each.

This combination, so often used, when put together in the above proportions, is very liable to explode, especially if warmed.

6. Soda chlo., 2 dr.; antimon. sulph. aurat., 20 grs.

This combination, if even gently triturated, is liable to inflame with a crackling noise.

7. Lac. sulphuris, 3 grs.; antimon. sulph. aurat., ½ gr.; zinci valer., 2 grs.; potass. chlor., 2 grs. M. Ft. Pulvis. Make 10 alike.

Potash permanganate, when associated with any readily oxidizable substance, such as glycerine, explodes.

8. Chromic acid, 10 grs.; glycerin, 1 dr.

This mixture is liable to explode, unless the glycerine is added to the acid drop by drop.

Iodine and ammonia form a very powerful explosive agent when combined, unless some water is introduced into the mixture, which seems to retard the development of nitrogen iodide, upon which the explosive properties depend.

9. Iodine, ʒss.; linim. camph. co.; linim. saponis, aa. ℥ii. M. Ft. Linim.

This combination exploded once in the hands of the pharmacist from the iodine and the ammonia in the liniment. camp. co. coming in contact.

10. Acidi nitric; acidi muriatici; tr. nucis vom., aa. ʒij. M.

This prescription was once ordered by a physician, and exploded after several hours.

11. Acid nitro-mur., ℥j.; tr. cardamomi, ℥ss. M.

Also this combination was the result once of a serious injury.—*Pacific Medical Journal.*

Headaches in Children.—Headaches are often hereditary; they have attacked children of the same family who have been brought up at a distance from one another, and whose surroundings have been quite different. In such cases there is something peculiar in the nervous system itself—a tendency to nervous disease. It will, I think, be often found on inquiry that the parents of such children are liable to nervous disease, nervous exhaustion, paralysis, etc., and perhaps some children of the family have had epilepsy, chorea, or asthma.

In many instances, too, there is some faulty condition of the blood. The brain badly nourished, through a scanty supply of blood, and that poor in quality, loses its balance, and cannot resume its tone.

I will now briefly allude to some of the varieties of headache in children. *Neuralgic* headache (one-sided headache) is not a very common type in children, but it oftener occurs than is generally supposed. So far as my experience goes, it has been met with chiefly among *three* classes of children. 1. Those of the neurosal temperament, whose nervous system is easily fretted, excited, and therefore sooner exhausted. If such children are pressed too much with their studies, then they the more readily suffer. Any degree of intellectual exertion is exciting to children of timid and delicate constitution, who are not only too anxious to learn, but cannot throw their studies off the mind. 2. Those children who have been reduced by some long and exhausting illness, in-door confinement and bad air. 3. Those born of delicate parents, and who are badly fed.

Neuralgic headache in connection with dental caries is by no means uncommon in children from six to twelve years of age. Whenever the headache is one-sided, the mouth should always be examined, for a decayed tooth may be keeping up the pain, and if so, cod-liver oil, iron, quinine, and arsenic, will be of no service.

Another class of headache is that which is *nervous* and *congestive.* The term "*nervo-hyperæmic*" was given to it by the late Dr. Symonds, of Clifton. It is a condition in which the cerebral vessels are over-loaded, and the nervous element is disturbed also. This form of headache is met with in children of both sexes, and is as much due to cerebral hyperæmia as to nerve disturbance. Shocks to the nervous system and overloading of the digestive organs are among the most frequent causes. The pain is, for the most part, confined to the forehead, and seldom seizes any other part of the head.

Headaches are common in rickety children, due to brain exhaustion, and to pressure from an increase of serum both in the lateral ventricles and between the convolutions. The head gets large, while the face appears to shrink. The children so suffering sleep badly, toss about at night, and are excitable and peevish during the day. Confinement to school and study, deficient or bad food, will often bring on a seizure by lowering the general health. Bromide and iodide of potassium, belladonna, etc., are useful in these cases. In the intervals of the attack cod-liver oil and pure air are not to be forgotten.—*W. H. Day, M.D., in Med. Press and Circular.*

---

NATIONAL JURISPRUDENCE.—It is the opinion of the writer that the whole matter of quarantine is within the competent jurisdiction of Congress; and this opinion is based upon judicial decisions.

It has been decided:

That the power of Congress to *regulate* commerce with foreign nations, or between the several States, is exclusive.

That to regulate, implies full power over the thing to be regulated.

That it may regulate ships and vessels so far as they are engaged in the carrying trade.

That it includes every subject of commerce to which legal discretion may apply; and to regulate the whole subject as much by what it leaves out, without any positive regulation, as by what it expressly provides for.

That it is for Congress to determine when its full power shall be brought into action, and as to the regulations and sanctions which shall be provided.

That the power may be exercised wherever the subject exists; and this may be within the territorial jurisdiction of the several States, and, when exercised, is exclusive of the same power in the several States.

That the power to regulate comprehends the control for that purpose, to the extent necessary, of all the navigable waters of the United States accessible from a State other than those in which they lie; to the control of all navigable waters extending to the sea-coast, and does not stop at the State boundary.

That in the concurrent powers of the National and State Governments, whilst the States are not absolutely prohibited from legislation as to regulating commerce, they may do so while the power of Congress lies dormant. Amongst the concurrent powers of Congress and the States, quarantine is enumerated; and it has been decided that, where Congress does not act, the States may legislate on local and appropriate matters, though they may be connected with commerce; but, in case of the action of Congress, the State laws must yield.

Such are the facts upon which the writer bases his opinion that both quarantine and all regulations flowing from it concerning the public health, so far as the States are concerned, are within the competent jurisdiction of the Congress.—*T. J. Turner, M.D., in Sanitarian.*

---

Small pox and Vaccination.—M. Greenwood reports some interesting cases, which occurred in a recent epidemic of small-pox, and which illustrate very forcibly the protective effect of vaccination.

1. A woman suffering from small-pox suckled her child throughout nearly the whole period of the disease. The latter, vaccinated successfully a month before, never showed any symptoms.

2. Small-pox occurred in a German family and the oldest of four children eventually succumbed to it. He was the only one in the family unvaccinated, and was nursed for nearly a week at home among the other three, all of whom escaped. The father believed in vaccination, but living in the United States at the time when this child was born, and vaccination being non-compulsory, he had neglected to have it done.

3. Small-pox broke out in a family of three children, none vaccinated. On the removal of the oldest, aged seven, to the hospital, the other two were vaccinated, though the mother said that the second child, aged five, was not feeling very well at the time. The vaccination was successful in both cases; but in that child, as the vaccinal pustules were beginning to die away, just ten days after vaccination, a slight but distinct variolous eruption broke out over the body. The unvaccinated child died of the disease; the other scarcely had any constitutional symptoms at all, the vaccine, owing to its shorter period of incubation and action, having anticipated the action of the variolous poison which was already in the system—*British Med. Journal.—Louisville Med. News.*

---

IMPURE VACCINE VIRUS.—The *Mail and Express* of New York contains an interesting article upon the quality of the vaccine virus, now finding such a ready sale in all parts of the country, from which it appears that there is now in the market, and in popular demand, an article which the patentee claims to be composed of "solid animal lymph made into a thick mass," to which has been given the name of "Cone." It is a small, cone-shaped mass of dark color, mounted upon a glass stand and covered with a cup of the same material. These cones retail for $3 00 apiece, and, as nearly 100 vaccinations can be made with one, this cheap virus is largely employed. It is, however, very impure, and has in some instances given bad results, such as erysipelas and painful sores; and in one case, viz.: that of J. W. Whittaker, Chief Engineer of the U. S. transport ship, Minnesota, it produced death.

Another form of the same thing, but which is only made and sold in New York, is vaccine powder. This is the result of reducing imperfect humanized crusts, secondary crusts, and, in fact, everything in the shape of a crust or scab resulting from vaccination except the perfect typical primary crust, to a powder, which is retailed by the grain. Such matter is necessarily of poor quality, and more or less adulterated.

We commend this matter to the attention of the State Board of Health, for if we have not a law which will deal with this most dangerous form of adulteration it is time we knew it, and if we have such a law it is time that it was being enforced.—*Sanitary Engineer.*

---

Puerperal Infection in the Male.—I see an extract from the *Centralblatt fuer Gynækologie* copied in several medical journals in regard to a case of puerperal infection of the male, in which a husband had intercourse with his wife after it was supposed she had entirely recovered from an attack resembling puerperal fever, which was followed in a few days by an attack of erysipelas of the penis resulting in gangrene of the parts and death on the sixth day, with well marked symptoms of septic poisoning. The case seems to have attracted considerable attention as being a very strange occurrence, and induces me to report to you a similar case, that I previously reported to the Athens County Medical Society in 1877. During an epidemic of puerperal fever which occurred in Nelsonville, a few years since, my brother, Dr. D. Lod. Gilliam, now of Colubus, O., and myself, were attending a colored woman who had an attack of what we diagnosed as puerperal fever. One morning after she had begun convalescing we noticed some bad symptoms, and on enquiry she told us that her husband had insisted on having intercourse with her, and she being too weak to resist he had accomplished his desires. She made a good recovery in course of time, but her husband was attacked two or three days after the occurrence we have related with erysipelas of the penis and scrotum, followed by gangrene and extensive sloughing of the parts, and died on the sixth day with well marked symptoms of septicæmia. He acknowledged to having had intercourse with his wife and attributed his disease to that. Some weeks before his sickness he had been under treatment for gonorrhœa, but I do not know whether he had recovered at the time of his sickness. If not, probably that might have been the cause of this infection.—*Chas. F. Gilliam, M.D., in Detroit Lancet.*

---

Chancroid.—The next case is that of a negro about twenty-five years old, who has, he says, a skin disease on the penis. There is an ulcer of the size of a large split pea, occupying the inner surface of the prepuce and its reflection on to the glans penis. There are also two or three small superficial ones, and a marked excoriation on the glans itself. The large ulcer has an elevated border, and no induration. It made its appearance as a small lesion, four or five days ago, which, he states, was about six days after intercourse. This is plainly a chancroid. You will find it stated that chancroids may appear five, seven, or more days, after infection. In my experience the sores usually make their appearance much earlier than this. I recall an exceptional case, seen recently in a physician, where an excoriation the size of a small split pea was developed within twelve hours after the infecting intercourse.

The treatment in this case will consist in his maintaining absolute cleanliness, by washing the parts three or four times a day, and then dusting with a small amount of iodoform powder. If the odor of the powder is too offensive, it may be made into an ointment with balsam of Peru and vaseline :—

℞.—Iodoformi,
Balsami peruvianæ, - aa. gr. xv.
Unguenti petrolei, - - gr. xxx. M.

Sig.—To be applied two or three times daily.

The ulcer will probably soon heal under this treatment.—*Clinic of Louis A. Duhring, M D., from Med. and Surg. Reporter.*

---

Treatment of Hydrocele.—J. S. Wight operates for Hydrocele as follows: The patient being anesthetized, a long, curved bistoury is introduced in front near the lower part, the point being brought out in front near the upper part, the tissue between the two points being readily divided by the edge of the bistuory. If the testicle is so diseased as to render it necessary, it is removed ; otherwise, it is left *in situ*. If gelatinous fibroid material is found about the testicle, it is carefully removed with a pair of scissors. The opened cavity is then washed out with a two per cent. carbolic acid solution and packed with oakum containing carbolized oil, which is kept in place by a proper bandage. This must be left for two or three days, or until more or less suppuration takes place, so that it can be easily removed without injuring the young granulations and without exciting bleeding. The wound is then dressed with oakum, carbolized or not, from day to day, the waste products being carefully removed, till scar tissue is formed and the sac obliterated, which ordinarily occurs in a few days.

Dr. Wight considers this operation as sure in its results, and safer than any method of treatment by the injection of irritating fluids in order to cause inflammation and adhesion.—*Med. and Surg. Rep.*

---

Surgical Triumphs.—Professor Nussbaum has just published a very interesting address, delivered on the Anniversary of the hundredth birthday of Philipp Franz Von Walther, who was born in 1782, and died in 1849; after an account of his life and work, Nussbaum makes a rapid survey of progress since Walther's day. Anæsthetics, antiseptics, and bloodless operations, are all advanced as surgical triumphs. Of ovariotomy, he says, "about 40,000 years of life have already been gained for women by successful ovariotomies;" and he believes that lives will be saved and much suffering prevented by Hegar's operation of removing the ovaries to anticipate the climacteric age in women the subjects of bleeding uterine fibroids. The cure of reflex epilepsy by nerve-stretching he regards as a great advance in therapeutics. Excision of a kidney or of the spleen, of parts of a cancerous bladder or prostate, of the rectum and of the pylorus, he also regards with confident hope of improving results; and he believes it "not quite impossible that diseased portions of lung may be successfully excised." Our German colleagues are certainly not behind us in courage and adventure.—*Brit. Med. Journal.—Canada Med. Record.*

---

Croupous Pneumonia.—1. Croupous pneumonia is an acute endemic disease, of zymotic origin.

2. The lung lesion is but a local manifestation of the constitutional disturbance, analogous to the ulceration of Peyer's glands in typhoid fever.

3. The specific cause remains undetermined; nor is the specific nature of the *cause* of any zymotic disease understood.

4. The time of year when the cause seems to be most active, includes the months of January, February, and March—although the disease occurs at all seasons.

5. The disease must run its course. Treatment should therefore be symptomatic and expectant.

6. It is urged that croupous pneumonia be given its proper position in the classification of diseases both in systematic treatises on the Practice of Medicine, and in the public mortuary reports of Boards of Health.—*D. W. Prentiss, M.D., in Trans. Am. Med. Asso'n.*

---

SEWERAGE.—The aim of the sewer engineer of the present day, guided by the study of the fundamental principles involved and the experience of the past, is to provide channels for conveying sewage to its destination with such rapidity and completeness that the transport shall be effected before the most dangerous stage of decomposition shall have set in. The prevention of sewer gas is aimed at by removing the sewage before it exhales the the gas. This demands the construction of channels with smooth surfaces, and true gradients, and the cleansing of all places where deposits may be apt to occur, systematically and frequently. The accumulation of such gases as may be generated, must be prevented, by dissipating them as rapidly as formed, furnishing them with free access to the open, outer air, and preventing them from entering close apartments.

That is all there is to a perfect system of sewerage.

It is simple, plain and inexpensive. The more *gimcracks* and complicated contrivances there are applied to the work, the less perfect is the system.—*Sanitary Engineer.*

---

NERVE-STRETCHING.—From my own experience I can simply draw these conclusions:

1. That moderate stretching of the nerve produces merely a temporary motor paralysis, easily recovered from, and a very considerable paralysis of sensation, likewise easily recovered from.

2. That severe stretching produces a marked motor paralysis of long continuance (months), and a tolerably complete paralysis of sensation, much more quickly recovered from than is the motor paralysis. Cases of spasm should therefore be stretched vigorously.

3. That profound cutaneous anæsthesia may be removed for several months, and perhaps permanently.

4. I have been unable to observe, as has been claimed, that sensibility is relatively lost to a greater extent and more persistently than motion, either by moderate or severe stretching. In my cases motor paralysis has been more persistent than sensory.—*William J. Morton, M.D., in Journal of Nervous and Mental Diseases.*

---

THE MEDICAL STUDENT'S PRIMER.—What place is this? This is the Pathological Society. How does one know it is the Pathological Society? You know it by the specimens and the smells.

What does that gentleman say? He says he has made a post-mortem. All the gentlemen make post-mortems. They would rather make a post-mortem than go to a party.

What is that on a plate? It is a tumor. It is a very large tumor. It weighs one hundred and twelve pounds. The patient weighed eighty-eight pounds. Was the tumor removed from the patient? No, the patient was removed from the tumor. Did they save the patient? No, but they saved the tumor.

What is this in the bottle? It is a tapeworm. It is a long tapeworm; it is three-quarters of a mile long. Is that much for a tapeworm? It is indeed much for a tapeworm, but not much for the Pathological Society.—*N. Y. Med. Record.*

---

TREATMENT OF VARICOCELE BY EXCISION OF REDUNDANT SCROTUM.—R. J. Levis calls attention to the method of treating varicocele, which was apparently first suggested and practiced by Sir Astley Cooper, but has never been generally adopted by the profession. He commends most emphatically the operation as performed with the aid of a clamp devised by Dr. M. H. Henry, of New York. The clamp is applied so as to include the integumental tissues, and the redundant portion is cut away with scissors or knife. Sutures may be introduced before or after the excision of the tissue, but must always be introduced before removing the clamp. A simple carbolized dressing is all that is necessary, and the results of this treatment have been extremely satisfactory, both to Dr. Levis and Dr. Henry.—*Phil. Med. Times.*

---

Colles' Views Respecting Syphilis.—Dr. McDonnell (*London Med. Record*), gives the following as Colles' principal discoveries in syphilis:

1. He establishes the opinion that secondary symptoms are capable of propagating the venereal disease.

2. While showing that a child may receive the infection of syphilis by sucking a nurse affected with secondary symptoms, he cannot recollect a case in which the diseased nurse infected the child unless she had an ulceration of the nipple.

3. He makes the important generalization (which Hutchinson has termed Colles' law), that a father cannot transmit syphilis to his offspring without implicating its mother.

Colles also draws a distinction between the local ulcer and the infecting chancre, contrary to the teaching of Hunter and other authorities of the period.—*Detroit Lancet.*

---

The Ethics of New York.—The proposition of the society of the State of New York, to hold consultations with all legally-qualified practitioners of medicine, does not exclude the licensed cancer quack, the midwife, and the chilropodist. It embraces all the *pathies.* Now, this is called ethics, and we are plainly informed by the *Record* that this is reform. Fortunately, the provisions for enforcing this code of defiance to all ethics and common decency are limited to the prostitutes of professional morals, and the country may yet be saved.—*Med. Herald.*

Iodine in Malaria.—In the *Maryland Medical Journal*, for February 15, 1882, Dr. R. B. Morison reports two hundred and fifty cases of malaria treated with tincture of iodine in fifteen minim doses, largely diluted, a quarter hour before meals. His cases show an even more favorable result than where cinchonidia is employed, and he states that iodine is now the routine treatment in all cases of acute malaria coming to the dispensary. Similar observations are reported in the *Indian Medical Gazette*, for January, by Baboo-Brojendry Nath Bamerjee at the meeting of the Calcutta Medical Society. He states that since 1878 he has made large use of this method of treatment, and in 1879 published in the Calcutta *Medical News* that he had cured ninety per cent. of five hundred cases. Since then, however, he has been led to distrust the reliability of his results, and believes that many of his earlier trials were made in cases which recover spontaneously, while others of these cases last, even under no treatment, only one day. Under such circumstances any drug will appear to act like magic. His present belief is that iodine will prove serviceable in about half the cases. The same author also reports cases in which *burnt alum* appeared to be of service.—*Med. News*.

---

A Curious Misuse of Terms.—A correspondent from Appleton, Wis., sends us the following amusing and suggestive note: "The vast amount of medical knowledge unexpectedly displayed by the non-professional press during the illness of the late President is now to a great extent a matter of record. As evidence of the singular accuracy of much of this knowledge, I offer the following statements, all of which were taken from the columns of leading daily papers of several large cities: 'Hyparenthesis' was feared, as also was 'hypodralic congestion.' Among the glands affected during the course of the case were the 'salwary conglomerated,' the 'sympathotic,' the 'partoid,' 'parotidid,' 'paratodit,' one of which, it was feared, would 'sulphurote,' while in the case of another, 'supperation' was the expected catastrophe. There were also 'metastalic abcesses,' and others which were 'metallastic' in character, and the 'plexus cosiacus' played an important part, which was also true of 'septicemid' and 'septicemæmia,' 'sceptic matter' was treated by the use of 'permanganesed potash' and 'permanganose potash,' while the vital powers were sustained by the use of 'reptonized enemetics,' and 'liquor nourishment.' Finally, Dr. Bliss gave a reporter his personal assurance that 'President Garfield had not been under the influence of optics for more than a week.'"—*Medical Record.*

---

SURGICAL MILLENIUM.—Mr. Spencer Wells has completed his one thousand cases of ovariotomy. Seven hundred and sixty-nine of these recovered. In the earlier part of his career, the mortality was 34 per cent.; later, it fell to 3 per cent.

---

THE next International Congress, which is to meet in 1833, at Copenhagen, has already begun its organization. Professor Panum has been nominated President, and Carl Lange secretary.

---

REFERRING to medical expert testimony, Guiteau says: "If I had the money I could get fifty of the best experts in the country to swear that I was as crazy as a loon." Who doubts it?—*Detroit Lancet.*

---

DR. HORATIO R. BIGELOW, of Washington, D. C., is preparing a paper on ovariotomies performed in America, and will be glad to receive full reports of all such cases.

---

DR. E. A. ADAMS, one of the medical officers of the Asylum for the Insane in Michigan, was fatally stabbed, in January, by one of the inmates.

